# Caspase-11 and NLRP3 exacerbate systemic Klebsiella infection through reducing mitochondrial ROS production

**DOI:** 10.3389/fimmu.2025.1516120

**Published:** 2025-02-17

**Authors:** Yuqi Zhou, Zhuodong Chai, Ankit Pandeya, Ling Yang, Yan Zhang, Guoying Zhang, Congqing Wu, Zhenyu Li, Yinan Wei

**Affiliations:** ^1^ Department of Pharmaceutical Sciences, Irma Lerma Rangel School of Pharmacy, Texas A&M University, College Station, TX, United States; ^2^ Department of Physiology, College of Medicine, University of Kentucky, Lexington, KY, United States

**Keywords:** *Klebsiella*, sepsis, pneumonia, inflammasome, caspase-11, NLRP3, ROS

## Abstract

**Introduction:**

*Klebsiella pneumoniae* is a Gram-negative bacterium and the third most commonly isolated microorganism in blood cultures from septic patients. Despite extensive research, the mechanisms underlying *K. pneumoniae*-induced sepsis and its pathogenesis remain unclear. Acute respiratory failure is a leading cause of mortality in systemic *K. pneumoniae* infections, highlighting the need to better understand the host immune response and bacterial clearance mechanisms.

**Method:**

To investigate the impact of *K. pneumoniae* infection on organ function and immune response, we utilized a systemic infection model through intraperitoneal injection in mice. Bacterial loads in key organs were quantified, and lung injury was assessed. Survival analysis was performed in wild-type (WT) and gene deficient mice. Mitochondrial damage and reactive oxygen species (ROS) production, as well as cytokine levels were measured in macrophages isolated from these mice to evaluate their contribution to bacterial clearance capacity.

**Results:**

Our findings demonstrate that *K. pneumoniae* systemic infection results in severe lung injury and significant bacterial accumulation in multiple organs, with the highest burden in the lungs. Deficiency of caspase-11 or NLRP3 led to prolonged survival, a reduction in pulmonary bacterial load, increased blood oxygen levels, and decreased IL-6 levels in the lungs compared to WT controls. Furthermore, caspase-11- and NLRP3-deficient macrophages exhibited elevated mitochondrial ROS production in response to *K. pneumoniae*, which correlated with more effective bacterial clearance.

**Discussion:**

These results suggest that caspase-11 and NLRP3 contribute to *K. pneumoniae*-induced sepsis by impairing mitochondrial function and reducing ROS production in macrophages, thereby compromising bacterial clearance. The observed reduction in lung injury and increased survival in caspase-11- and NLRP3-deficient mice indicate that targeting these pathways may offer potential therapeutic strategies to improve host defense against systemic *K. pneumoniae* infection.

## 1 Introduction

*Klebsiella pneumoniae (K. pneumoniae)* is a major causal agent of sepsis and is often isolated in blood cultures from sepsis patients (1–3). *K. pneumoniae* infections are usually associated with high mortality rate, particularly in immunodeficient individuals (4). *K. pneumoniae* can invade multiple organs including the lung, blood, liver, and urinary tract, and is a leading cause of hospital-acquired pneumonias (5, 6). Furthermore, *K. pneumoniae* has been singled out by the World Health Organization as a public health threat due to the wide-spread antibiotic resistance among *K. pneumoniae* strains. Many *K. pneumoniae* clinical isolates are resistant to antimicrobials, especially the first line medicine including β-lactams, aminoglycosides, fluoroquinolones (7, 8). Thus, understanding of the mechanisms by which *K. pneumoniae* triggers host immune response is critical for the development of therapeutic strategies targeting *K. pneumoniae*-induced sepsis.

Several types of inflammasomes have been found to be involved in the host defense against microbial infection, and the two major ones are NLRC4 (NOD-like receptor family, CARD domain containing 4) and NLRP3 (NOD-like receptor family, pyrin domain containing 3) (9). While inflammasome activation during *K. pneumoniae* infection has been the focus for several studies, them all used the lung infection model, in which the bacteria were inoculated either intranasally or intratracheally (10–13). The NLRP3 inflammasome was shown to be important in promoting caspase-1 activation and IL-1β release in murine macrophages following *K. pneumoniae* challenge (11). However, the contribution of NLRC4 to the pathogenesis of *K. pneumoniae* infection is controversial. Willingham et al. reported that deficiency of NLRC4 had little impact on mouse survival following *K. pneumoniae* infection and did not affect lung histology, IL-1β secretion, and lung bacterial load, arguing against a role of NLRC4 in host immune response to *K. pneumoniae* infection (11). In contrast, Cai et al. reported that NLRC4 plays an important role for host immunity in lungs following *K. pneumoniae* infection (10). *K. pneumoniae* was found to induce caspase-1 activation and secretion of IL-1β from macrophages through the NLRC4 pathway, but pyroptosis did not occur as GSDMD cleavage was not observed. Inflammasome activation as a host defense mechanism during *K. pneumoniae* infection originated from the intestine has not been explored. The gastrointestinal colonization of *K. pneumoniae* serves as a major reservoir for transmission and infection to other sites (14), and a high level of association between gastrointestinal carriage and subsequent infection by a patient’s own *K. pneumoniae* strains has been reported (14, 15). Here we used the systemic infection model via i.p. injection, through both *in vivo* and *in vitro* experiments, to explore the mechanism of inflammatory responses during *K. pneumoniae* systemic infection.

## 2 Materials and methods

### 2.1 Mice

Wide-type (WT) C57BL/6J, caspase-1^-/-^, caspase-11^-/-^, NLRP3^-/-^, NAIPs^-/-^, TLR4^-/-^, GSDMD^-/-^/GSDME^-/-^ and caspase1^-/-^/caspase 8^-/-^/Ripk3^-/-^ mice were housed in the Animal Care Facility at Texas A&M University, School of Pharmacy, following institutional and National Institutes of Health guidelines after approval by the Institutional Animal Care and Use Committee. Male mice of age 8-12 weeks were used in all experiments.

### 2.2 Bacteria

*K. pneumoniae* (ATCC 43816) was grown in LB at 37°C to the log phase, collected, allocated, and stored at -80°C. Bacteria number was quantified using plate-based colony counting method.

### 2.3 Survival assays

Wide-type (WT) C57BL/6J and different gene knockout strains of C57BL/6J were infected with bacteria through intraperitoneal injection of 1×10^6^ CFU (or the indicated amount) in 200 μL sterile PBS. The mice were then left with ad libitum food and water and observed over time to monitor their activity and health deterioration symptoms.

### 2.4 Prothrombin time and plasma thrombin-antithrombin

Blood was collected from tribromoethanol (Avertin)-anesthetized mice by cardiac puncture with a 23-gauge needle attached to a syringe pre-filled with 3.8% trisodium citrate as anticoagulant (final ratio at 1:7). Blood was centrifuged at 1500 g for 15 minutes at 4°C to obtain plasma. PT was determined with Thromboplastin-D (Pacific Hemostasis, Cat#100357) in a manual setting according to manufacturer’s instruction, using CHRONO-LOG #367 plastic cuvette. Plasma TAT concentrations were determined using a mouse TAT ELISA kit (Abcam, Cat#ab137994) at 1:50 dilution according to the manufacturer’s instructions.

### 2.5 *In vivo* inflammation study

Bacteria were resuspended and diluted in sterile PBS. Mice were injected i.p. with 1 × 10^7^ CFU of *K. pneumoniae* in 200 μL sterile PBS. Blood samples were collected before or at various times (90 minutes, 4 hours, and 6 hours) following injection via retro-orbital bleeding in an EDTA tube and were centrifuged at 10,000 g for 1 minute at room temperature to obtain plasma. IL-1β, IL-6, and TNFα were measured using ELISA kit (Invitrogen) following manufacturer’s instruction.

### 2.6 Blood oxygen saturation

Bacteria and LPS were resuspended and diluted in sterile PBS. Mice were injected i.p. with 1×10^7^ CFU of bacteria or LPS (10 mg/kg body weight) in 200 μL sterile PBS. Each mouse was anesthetized using 2.5% Tribromoethanol to facilitate placement of a PawClip Sensor and allowed to acclimatize for 5 minutes. Arterial O_2_ saturation level was measured continuously using PhysioSuite (Kent Scientific, USA) in accordance with manufacturer’s instructions. Measurements were recorded every 1 hour after bacteria or LPS injection, up to 7 hours.

### 2.7 Blood chemistry measurement

AST, BUN, Amylase, CK-MB, and LDH were measured by Rodent Preclinical Phenotyping Core at Texas A&M University. To compare the organ damage caused by bacteria, mice were i. p. injected with 1×10^7^ CFU of *K. pneumoniae*. 300-500 μL of blood samples were drawn from heart into heparinized syringe at 6 hours post infection. Plasma samples were collected after centrifugation.

### 2.8 Histologic examination

Histological samples were prepared by Veterinary Medicine & Biomedical Sciences Core Histology Lab at Texas A&M University. Mice were injected i.p. with 1×10^7^ CFU of *K. pneumoniae* in 200 μL sterile PBS. Lungs were perfused and isolated at 6 hours after injection of bacteria. Lungs were fixed in formalin for 2 days and then embedded in paraffin. Sections were cut at 4 μm and stained with hematoxylin and eosin (H&E). Images were captured with an ECHO Rebel Hybrid microscope (ECHO, USA). For histological analysis, lung sections were examined and scored as described (16). In short, the following parameters were scored on a scale of 0 (absent), 1 (mild), 2 (moderate), 3 (severe), and 4 (very severe): interstitial damage, vasculitis, peribronchitis, edema, thrombus formation, and pleuritis. Total lung pathology score was expressed as the sum of the scores for each parameter.

### 2.9 Bacterial burden in key organs

Bacteria were resuspended and diluted in sterile PBS. Mice were injected i.p. with 5×10^6^ CFU of bacteria in 200 μL sterile PBS. Whole heart, lung, liver, spleen and kidney were harvested and homogenized on ice in 9 volumes of sterile saline. Serial dilutions in sterile saline were made from these homogenates, and 100 μL were plated onto agar plates and incubated at 37°C. CFUs were counted after overnight incubation. MitoQ (MedKoo Biosciences, Morrisville, NC), a mitochondria-specific antioxidant, was administered to mice via i.p. injection at a dose of 0.1 mg per mouse 48 hours prior to bacterial injection when indicated.

### 2.10 Cytokines in lung tissues

Lung homogenates were lysed in 2× RIPA buffer (312 mM NaCl, 2 mM EGTA, 2% Triton X-100, 2% sodium deoxycholate, 0.2% SDS, 20 mM Tris, pH 7.4), containing freshly added protease inhibitor cocktail (PI8340) and spun at 1,500 g at 4°C for 15 minutes. The supernatant was used in cytokine measurement. IL-1β, IL-6, and TNFα were measured using ELISA kit (Invitrogen) following manufacturer’s instruction.

### 2.11 Macrophage depletion

Mice were injected retro-orbitally with 40 mg/kg of liposomal clodronate (Encapsula NanoSciences, Nashville, TN) at 24 h before being challenged with *K. pneumoniae*. The same amount of plain liposomes was injected in the control mice.

### 2.12 Isolation of bone marrow derived macrophages

Macrophages were isolated as described (17). BMDMs were cultured for 5 days in the presence of 15% conditional medium from L929 cells.

### 2.13 *In vitro* infection of BMDMs

Isolated BMDMs in DMEM medium were plated in either 24 well plate or 12 well plate at a density of 1×10^6^ cells/mL. The BMDMs were then allowed to attach to the plates by incubating at 37°C and 5% CO_2_ overnight. For mitochondrial ROS measurement, the medium was changed to Opti-MEM low serum medium. *K. pneumoniae* was resuspended in sterile PBS and added to cells at 50 MOI and incubated for 90 minutes at 37°C and 5% CO_2_ to allow for bacterial internalization. Next, macrophages were washed with sterile PBS three times and the indicated medium containing 300 μg/mL gentamicin was added to kill extracellular bacteria. The medium containing gentamicin was refreshed every 90 minutes. For the treatment of the ROS scavenger NAC (Sigma), NAC was added to a final concentration of 20 mM together with gentamycin. For the treatment with the mtROS inhibitor MitoQ, BMDMs were incubated in DMEM medium containing 1 µM mitoQ for 1 hour prior to *K. pneumoniae* infection. MitoQ was refreshed every 90 minutes together with gentamycin as well.

### 2.14 Immunoblotting

For immunoblotting, 1×10^6^ cells were pre-stimulated with 1 μg/mL Pam3CSK4 for 5 hours before *K. pneumoniae* (50 MOI) or LPS (2 μg/mL and Fugene (Promega Cat#E2311)) were added. For *K. pneumoniae* treated sample, macrophages were washed with sterile PBS after 90 min and fresh medium containing 300 μg/mL gentamicin was added followed by an additional incubation of 6 hours. The LPS treated samples were also incubated for an additional 6 hours. Cell supernatants were then collected and subjected to TCA precipitation of proteins, whereas cell lysates were obtained by dissolving cells using SDS loading dye coupled with sonication. These samples were then used in immunoblotting to detect caspase-1, caspase-11 and IL- 1β. For immunoblotting of ASC, cell pellets were collected in 500 μL caspase buffer (10 mM PIPES (pH 7.2), 10% (w/v) sucrose, 100 mM NaCl, 0.1% (w/v) CHAPS hydrate and 1 mM DTT) containing freshly added 1% protease inhibitor cocktail (PI8340). After three freeze-thaw cycles, cells were centrifuged at 400× g for 5 minutes. The supernatant was collected and centrifuged at 15,000× g for 10 minutes to get the soluble and pellet samples. The pellet samples were resuspended in 100 μL PBS and 2 mM disuccinimidyl suberate (DSS) was added. The samples were incubated at room temperature for 30 minutes with rotation, and then subjected to centrifugation at 15000× g for 10 minutes. The pellets were dissolved in 100 μL SDS loading dye. Pro-caspase-1 and caspase-1-p20 were detected using anti-caspase-1(p20) antibody (Adipogen, Cat#AG-20B-0042) at 1:1000 dilution. Pro-IL-1β and IL-1β (p17) were detected using anti-IL-1β antibody (GeneTex, Cat#GTX74034) at 1:1000 dilution. Pro-caspase-11 and caspase-11 (p26) were detected using anti-caspase-11 antibody (Novusbio, Cat#NB120-10454) at 1:1000 dilution. ASC and its oligomer were detected using anti-ASC/TMS1 antibody (Cell Signaling, Cat#67824). β-actin and TOM20 were detected using anti-β-actin antibody (Cell Signalling, Cat#4970) and anti-TOM20 antibody (Proteintech, Cat#11802-1-AP) respectively at 1:1000 dilution. Blots were imaged using BIO-RAD ChemiDoc MP imaging system as well as Azure imaging system.

### 2.15 Intracellular bacterial burden

The BMDMs were plated at a density of 1×10^6^ cells/mL and changed to RPMI 1640 medium (supplemented with FBS, HEPES and L-glutamine). *K. pneumoniae* (50 MOI) was added into BMDM for 90 min, and then medium was changed and gentamicin added as described above. At 90 minutes and 6 hours after the addition of gentamicin, BMDMs were washed with sterile PBS three times and lysed using sterile RIPA buffer for 5-10 minutes at room temperature. Lysates were diluted in PBS and plated on petri dishes containing Brucella Broth Agar. The petri dishes were incubated at 37°C overnight to allow the growth of colonies, which was counted the next morning.

### 2.16 Flow cytometry

BMDMs were detached using 0.1 mM EDTA in PBS, collected in PBS and incubate with 25 μM MitoSox Red for 30 minutes or 200 nM TMRM for 15 minutes at 37°C to detect mtROS or mitochondrial membrane potential using flow cytometer.

### 2.17 Live cell imaging

BMDM were incubated with *K. pneumoniae* (50 MOI) for 90 min, before medium was changed and gentamycin added. After additional 6 hours, Mitotracker Deep Red FM (MTDR, red), Mitotracker Green (MTG, green), and Hoechst (blue) were added to final concentrations of 200 nM, 200 nM, and 1 µg/mL, respectively, followed by incubation of 30 min. Cells were then washed, covered with fresh DMEM medium, and imaged using LSM780 confocal microscopy with 63x oil immersion lens. Measurement of MTDR and MTG mean fluorescence intensity (MFI) was conducted using the software FIJI. Fifty cells were analyzed per sample to determine the MFIs of the MTDR channel and MTG channel, and the MTDR/MTG MFI ratio was determined.

### 2.18 Statistical analysis

All the data shown were represented as mean ± SEM. Significance was analyzed by either one-way or two-way analysis of variance (ANOVA), or non-paired t-test. Specific details about the asterisks presented above the bar or group of bars and significance measurements are presented in the figure legends. Each group of experiment contains at least four individuals, and each experiment was repeated at least three times.

## 3 Results

### 3.1 *K. pneumoniae* led to quick mouse death in the intraperitoneal injection model

**Figure 1:**
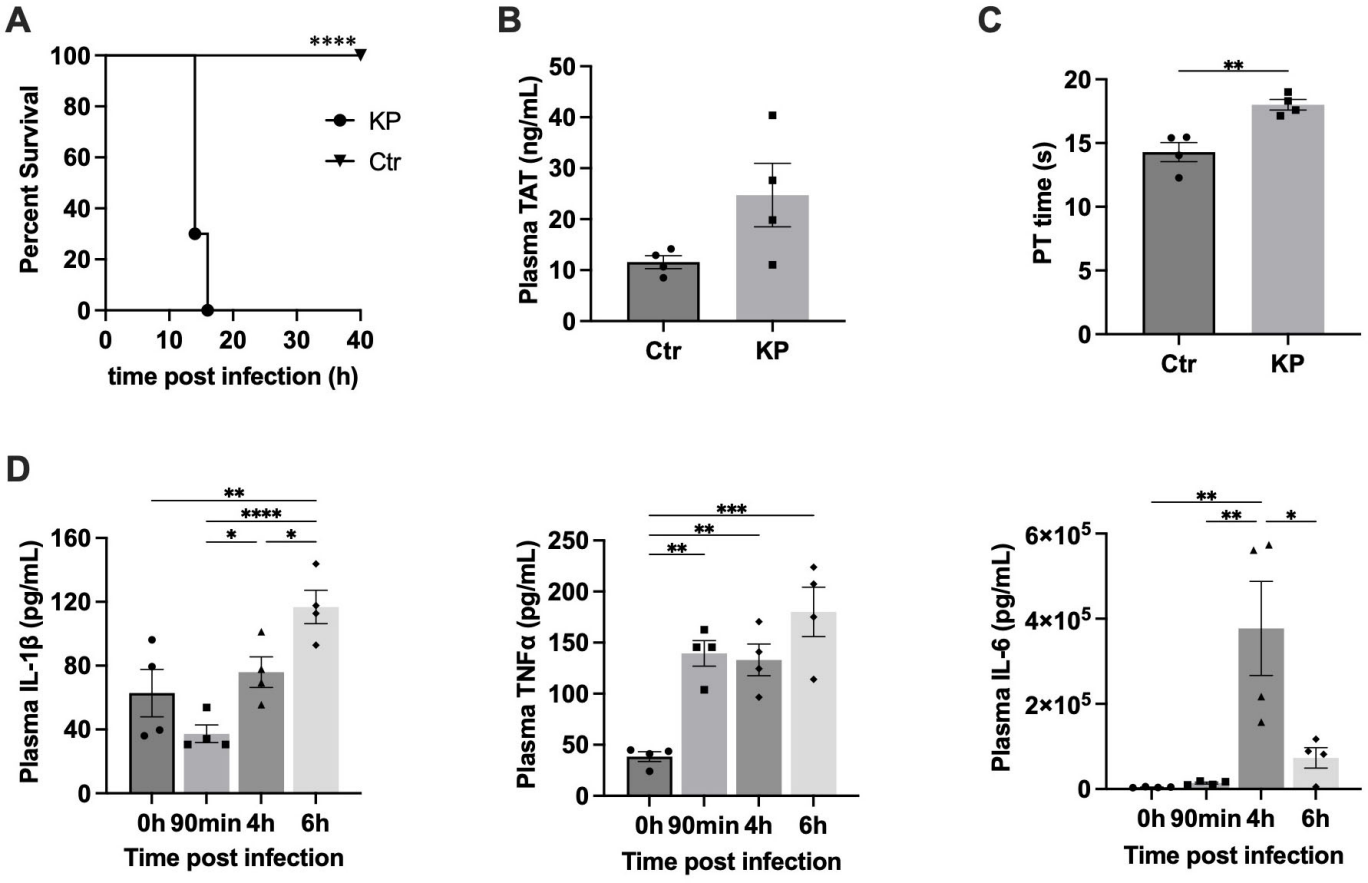
**(A)** *K. pneumoniae* (KP) systemic infection quickly killed mice. C57BL/6J mice were injected (i.p.) with PBS (Ctr) or bacteria (1×10^6^ CFU per mouse). Kaplan-Meier survival plots for mice are shown. n = 10-12 per group. ****p<0.0001 between KP treatment group and control group by log rank test [Mantel-Cox]. **(B, C)** Systemic *K. pneumoniae* (KP) infections induced blood coagulation. C57BL/6J mice were i. p. injected with PBS (Ctr) or KP (1×10^7^ CFU). Blood was collected at 90 min after injection. n = 4 for all experimental groups. **(B)**, Plasma TAT concentrations. **(C)**, Prothrombin time. Error bars denote SEM; *p<0.05, **p<0.01 by non-paired t-test. **(D)** Cytokine release induced by *K. pneumoniae*. Blood samples were collected at the indicated times after i. p. injection of *K. pneumoniae* (1×10^7^ CFU per mouse), and plasma cytokine levels were measured using ELISA. Error bars denote SEM; n = 4 for all experimental groups. *p<0.05, **p<0.01, ***p<0.001, ****p<0.0001 by non-paired t-test.

Since *K. pneumoniae* often causes systemic infection and sepsis (1–3), we utilized an i.p. injection model to investigate the mechanism of *K. pneumoniae* pathogenesis. We found that 1 × 10^6^ CFU of *K. pneumoniae* (strain ATCC 43816) killed all mice within 24 hours after injection (**Figure 1A**). We have recently reported that inflammasome activation and subsequent pyroptosis trigger coagulation (DIC), which plays a critical role in sepsis-associated lethality (18). We investigated whether the mortality rate associated with *K. pneumoniae* infection was due to severe coagulation. The prothrombin time (PT) and plasma thrombin-antithrombin (TAT) levels, two biomarkers for DIC, were measured (**Figure 1B, C**). Under the current experimental condition, challenge with *K. pneumoniae* led to prolonged PT time, but not statistically significant elevation of the TAT levels, suggesting mild DIC (19–21).

It has been well-established that severe inflammation is another common lethal complication of sepsis. We measured the plasma concentrations of the proinflammatory cytokines including IL-1β, TNFα, and IL-6 immediately before, or at 1.5, 4, or 6 hours after injection (**Figure 1D**). The plasma levels of these cytokines all increased significantly after injection with *K. pneumoniae*. IL-1β levels increased over time. TNFα level peaked at 1.5 h and remained plateaued at 4 and 6 hours post infection. The levels of IL-6 induced by *K. pneumoniae* peaked at 4 hours and quickly reduced to the basal level at 6 hours post injection. These findings suggest that *K. pneumoniae* induced systemic inflammation in mice.

### 3.2 *K. pneumoniae* challenge led to acute lung injury in systemic infection model

**Figure 2:**
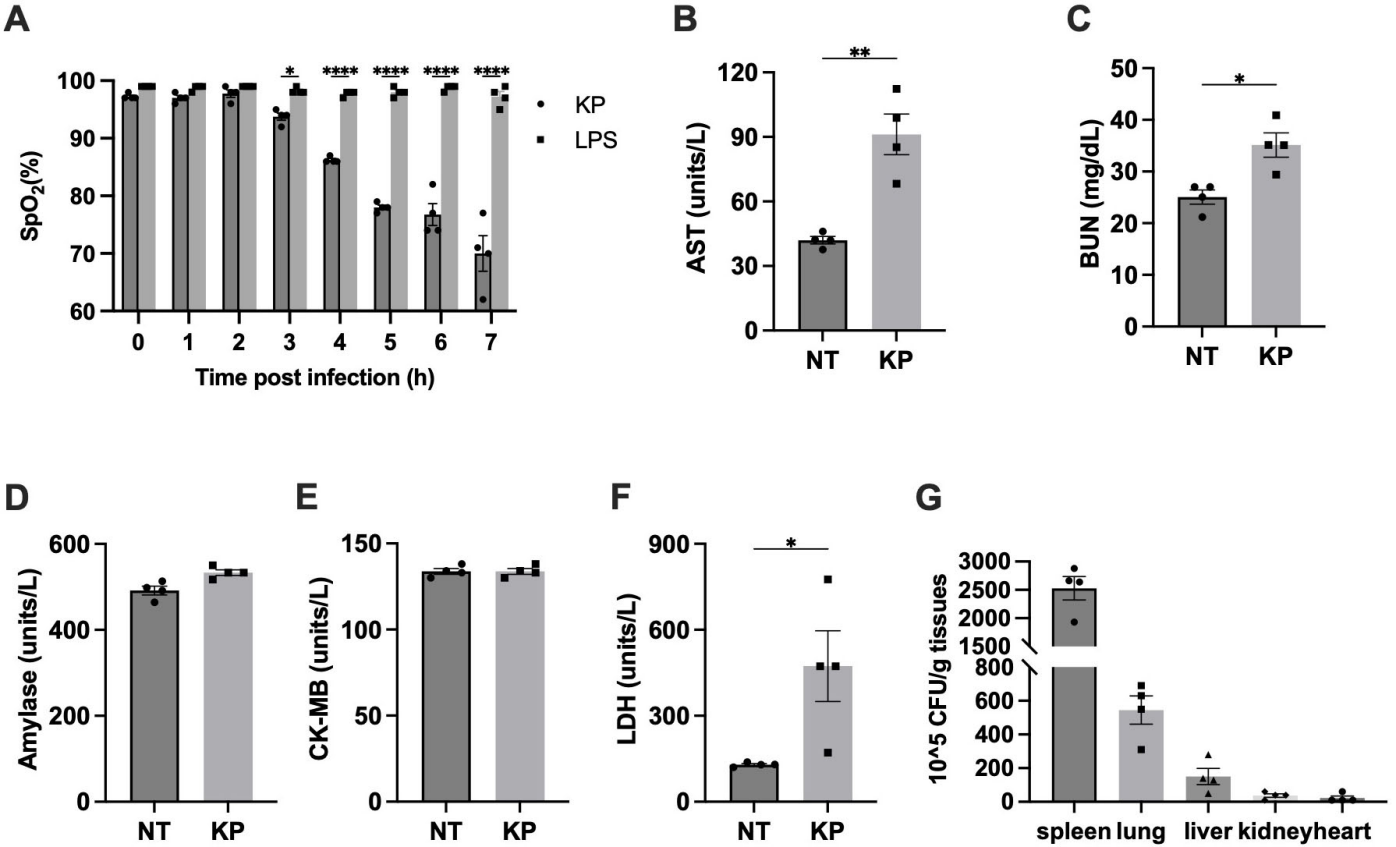
*K. pneumoniae* (KP) induced acute lung injury in systemic infection model. **(A)** SpO_2_ levels dropped dramatically in *K. pneumoniae*-treated mice. Mice were i. p. injected with 1×10^7^ CFU of *K. pneumoniae* (KP) or LPS (10 mg/kg body weight). SpO_2_ was recorded every hour after injection, up to 7h. Error bars denote SEM; n = 4 for all experimental groups. *p<0.05, **p<0.01, ****p<0.0001 between KP and LPS treatments by one-way ANOVA analysis. **(B-F)** Blood samples were collected at 6 hours after i.p. injection of PBS (NT) or the indicated bacteria (1×10^7^ CFU per mouse), and plasma AST **(B)**, BUN **(C)**, amylase **(D)**, CK-MB **(E)**, and LDH **(F)** were measured using the Beckman Coulter DxC 700 AU chemistry analyzer. Error bars denote SEM; n = 4 for all experimental groups. *p<0.05, **p<0.01 between KP and control by non-paired t-test. **(G)** Bacterial loads in key organs during systemic infection. Mice were injected (i.p.) with 5×10^6^ CFU of *K. pneumoniae* (KP). After 6 hours, mice were sacrificed and bacterial loads in the lung were determined. Error bars denote SEM; n = 4 for all experimental groups.

Acute respiratory failure is another common lethal complication of sepsis. We next measure the arterial oxygen saturation (SpO_2_) in mice challenged with *K. pneumoniae*. SpO_2_ has been used extensively to evaluate and monitor patients in clinical settings, as low oxygen saturation or hypoxemia is associated with conditions or diseases involving pulmonary function (22). A resting SpO2 ≤ 95% has been found to correlate with chronic obstructive pulmonary disease (COPD) (23, 24) and has also been identified as a risk factor for postoperative pulmonary complications (25). Oxygen saturation has routinely been monitored as a readout of lung function in studies using mouse models (26–29). SpO_2_ levels reduced from 95% to ~70% within 7 hours after mice were i.p. injected with *K. pneumoniae* (**Figure 2A**). To evaluate the contribution from *K. pneumoniae* associated LPS, a lethal dose of LPS (10 mg/kg body weight) was injected (30), which did not cause significant reduction in SpO_2_ within 7 hours. This result suggests that acute lung injury by *K. pneumoniae* infection cannot be explained simply by *K. pneumoniae* shedding of LPS.

We next determined the damage of other organs using specific biomarkers in blood. Liver damage is characterized by elevated aspartate transaminase (AST), while kidney damage is often associated with an increase in BUN (blood urea nitrogen). Pancreatic damage can be indicated by an increase in amylase levels, and heart damage is recognized by elevated creatine kinase-MB (CK-MB). In addition, lactate dehydrogenase (LDH) is a non-specific marker of cell death. Blood samples were collected 6 hours post injection and the levels of the indicated biomarkers were measured (**Figure 2B-F**). Pancreas function biomarker amylase and heart function biomarker CK-MB weren’t significantly affected. Significant increases of AST and BUM levels upon infection were observed, suggesting liver and kidney damage. Similarly, LDH were notably elevated upon infection. Altogether, this analysis indicated that lung, liver, and kidney function, but not pancreas and heart function, were impaired in *K. pneumoniae* treated mice at 6 hours post infection.

### 3.3 *K. pneumoniae* accumulated in the lung and caused lung inflammation

High accumulation of *K. pneumoniae* in lungs has been reported when the bacteria were delivered locally to the lung (10, 11). To investigate potential differences in organ accumulation upon intraperitoneal delivery, bacterial load in organs was analyzed 6 hours after injection as described (31). As shown in **Figure 2G**, the bacterial load in the spleen was the highest among all organs tested, consistent with its role in filtering lymph fluid and blood to remove cellular waste, old or damaged blood cells, as well as pathogens. Beyond the spleen, *K. pneumoniae* accumulation in the lung was the highest, consistent with the significant disruption of lung function. Presence of *K. pneumoniae* in the liver and kidney is consistent with the observed disruption of liver and kidney function.

### 3.4 Inflammasome activation contributed to *K. pneumoniae*-induced lethality in mice

**Figure 3:**
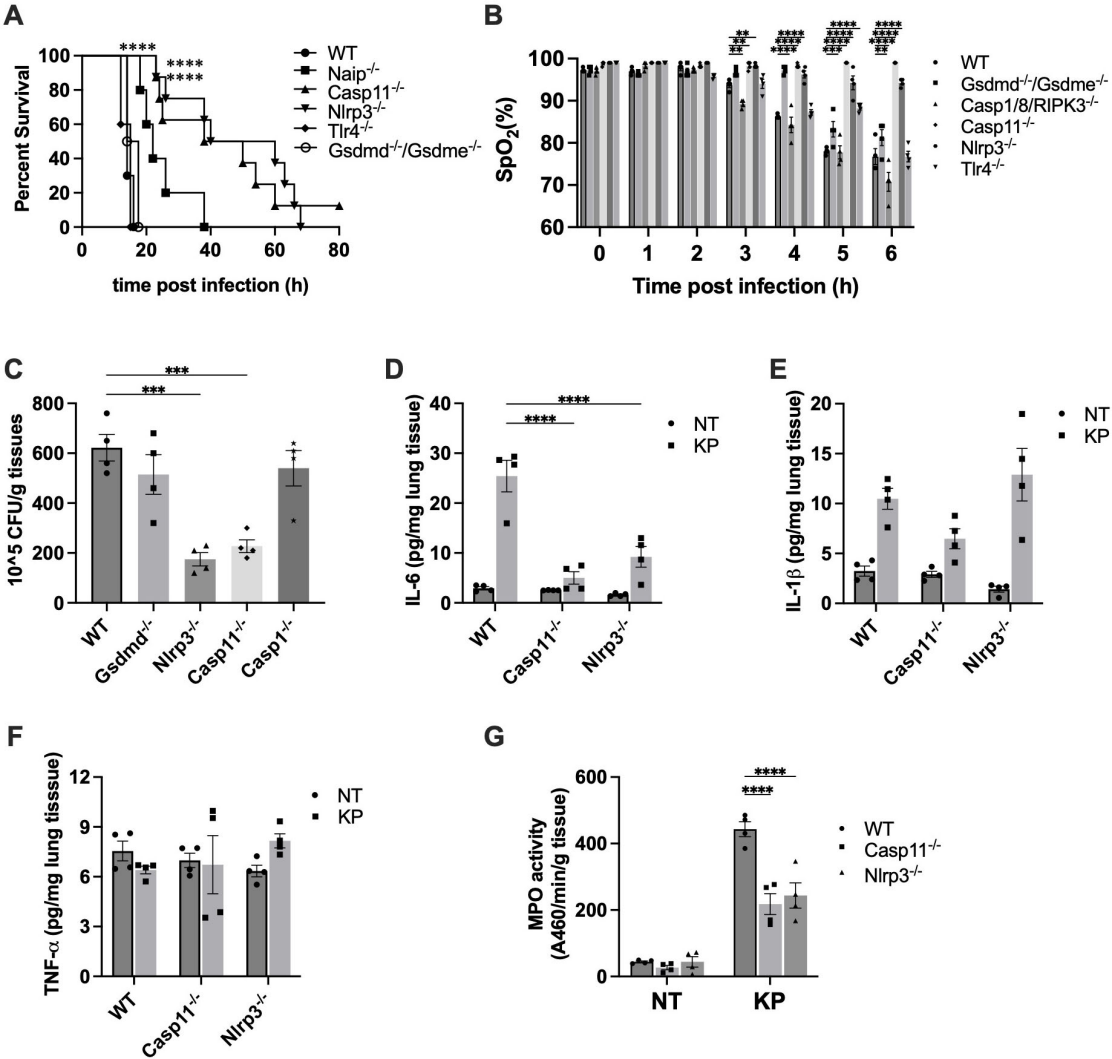
Inflammasome activation contributed to *K. pneumoniae* pathogenesis. **(A)** *K. pneumoniae*-induced lethality in the systemic infection model. Mice were injected (i.p.) with *K. pneumoniae* (1×10^6^ CFU per mouse). Kaplan-Meier survival plots for mice are shown. n = 10-12 per group. ****p<0.0001 between WT and deficient mice by log rank test [Mantel-Cox]. **(B)** *K. pneumoniae*-induced acute lung injury in systemic infection model was protected by caspase-11 and NLRP3 deficiency. Mice were injected by i.p. with *KP* (1×10^7^ CFU per mouse). SpO_2_ was monitored using infrared pulse oximetry. Error bars denote SEM; n = 4 for all experimental groups. **p<0.01, ***p<0.001, ****p<0.0001 between WT and deficient mice by two-way ANOVA analysis. **(C)** Caspase-11 and NLRP3 deficiency reduced bacterial load in lungs following *K. pneumoniae* infection. Mice were injected intraperitoneally with 5×10^6^ CFU *K. pneumoniae*. After 6 hours, mice were sacrificed and bacterial loads in the lung were determined. n=4 for all groups. ***p<0.001 between the WT and deficient mice by one-way ANOVA analysis. **(D-F)** Pulmonary cytokine levels in WT, Caspase-11 and NLRP3 deficient mice injected with *K. pneumoniae*. Lung tissues were collected and homogenized 6 hours after i.p. injection with the indicated bacteria (5×10^6^ CFU), and proinflammatory cytokines were measured by ELISA. Error bars denote SEM; n = 4 for all experimental groups. ****p<0.0001 between the WT and deficient mice by two-way ANOVA analysis. **(G)** Myeloperoxidase (MPO) activity in lungs of WT, Caspase-11 and NLRP3 deficient mice injected with *K. pneumoniae*. Mice were injected by i.p. with KP (1×10^7^ CFU per mouse). MPO activity of lung tissue was qualified (n = 4 per group) after 6 hours. ****p<0.0001 between the WT and deficient mice by two-way ANOVA analysis.

To investigate the signaling pathway underlying *K. pneumoniae*-induced lethality, we examined the survival of C57BL/6J and knockout mouse strains deficient in NAIP1-6, caspase-11, NLRP3, TLR4 or GSDMD/E after *K. pneumoniae* infection. All WT mice died within 20 hours after injection (**Figure 3A**). TLR4 serves as the cell surface receptor for LPS. Disruption of TLR4 had no discernible impact on survival. GSDMD and GSDME are two main executor proteins of pyroptosis. Depletion of both GSDMD and GSDME had no impact on survival, suggesting that pyroptosis is not a major mechanism contributing to *K. pneumoniae*-induced lethality. Deletion of caspase-11 and NLRP3 exhibited the most significant protection. Deletion of NAIPs resulted in a modest increase in the survival time.

### 3.5 Deficiency of caspase-11 and NLRP3 protected against *K. pneumoniae*-induced acute lung injury

Next, we investigated whether the prolonged survival of caspase-11 and NLRP3 deficient mouse was attributed to protection from acute lung injury. We measured SpO_2_ levels in WT mice and strains deficient in caspase-11 or NLRP3. Indeed, deficiency in the caspase-11 or NLRP3 largely protected against acute lung damage (**Figure 3B**). To determine whether other mechanism might also be involved in *K. pneumoniae*-induced lung injury, we measured SpO_2_ levels in mice deficient in caspase-1/8/RIPK3, TLR4 or GSDMD/E. The SpO_2_ levels of these mice strains were similar to that in WT mice, suggesting a minimal contribution from mechanisms involving these proteins. These results align with the observations from the survival studies, wherein disruption of caspase-11 or NLRP3, but not caspase-1, caspase-8, TLR4 or GSDMD/E, significantly extended mouse survival time.

### 3.6 The *K. pneumoniae* load in the lung was reduced in caspase-11 and NLRP3 deficient mice

As deficiency of caspase-11 or NLRP3 protected against *K. pneumoniae*-induced lung injury, we speculated the lung bacterial load to be reduced in caspase-11 or NLRP3 deficient mouse. To test this hypothesis, bacterial load in the lungs of WT, caspase-11^-/-^, and NLRP3^-/-^ mice was compared. Because NLRP3 activation normally leads to caspase-1 cleavage and activation, we also measured bacterial load in lungs of caspase-1^-/-^ mice. As shown in **Figure 3C**, *K. pneumoniae* load in the lung was markedly decreased in caspase-11 and NLRP3 deficient mice, compared to WT mice. Deficiency in caspase-1 or GSDMD did not have such an effect. Moreover, pulmonary IL-6 level upon infection significantly decreased in mice deficient in caspase-11 or NLRP3 (**Figure 3D**). Deficiency in caspase-11 or NLRP3 did not lead to significant reduction in the level of IL-1β nor TNFα in the lung tissues (**Figure 3E, F**).

**Figure 4:**
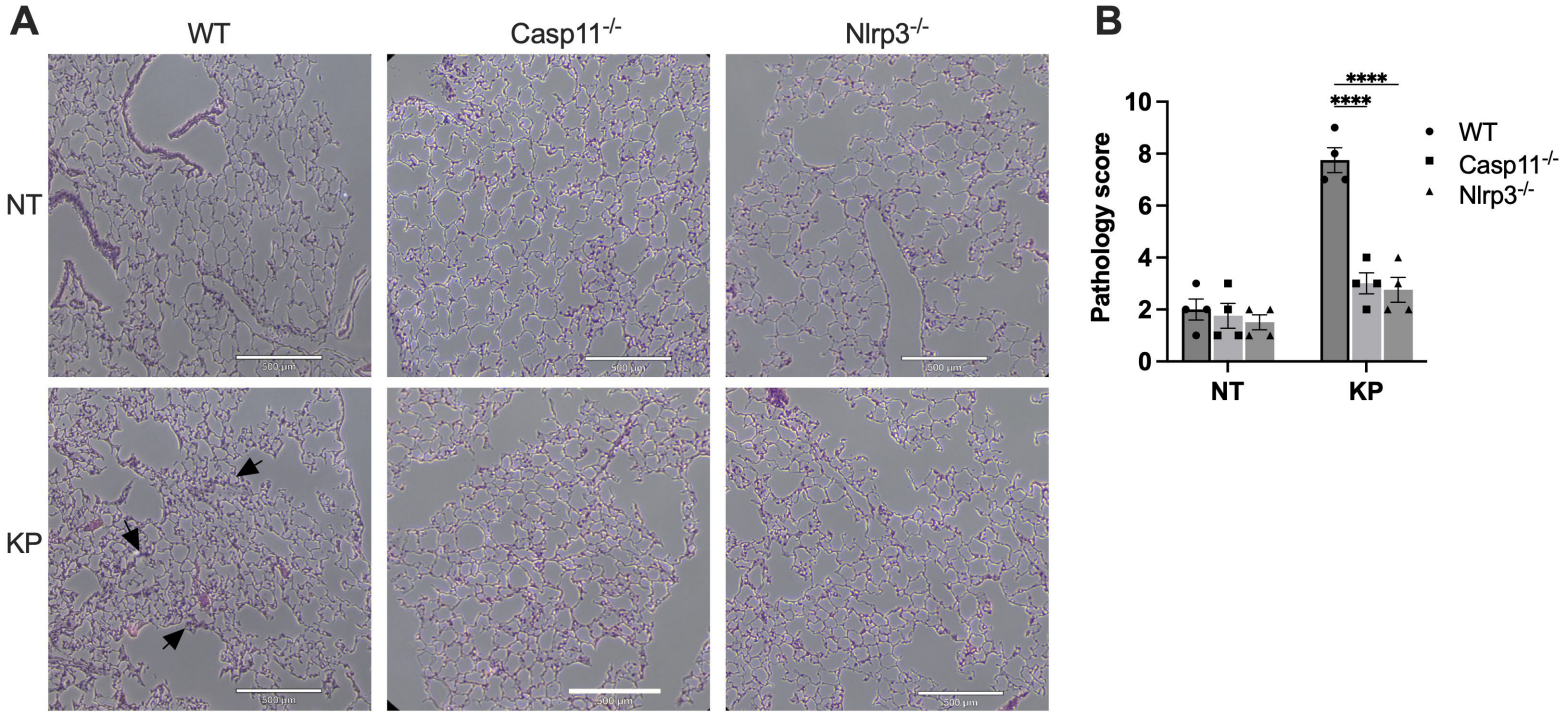
Histology of lungs from WT, caspase-11 or NLRP3 deficient mice infected with *K. pneumoniae* (KP). **(A)** Mice were injected by i.p. with *KP* (1×10^7^ CFU per mouse) or PBS (NT). After 6 hours, mice were sacrificed, and lungs were collected. Sections of lungs were stained with H&E and images were captured. Scale bar, 500 μm. **(B)** Total pathology scores of the lung sections. ****p<0.0001 between the WT and deficient mice by two-way ANOVA analysis.

During lung infection, neutrophils are recruited and release a large amount of myeloperoxidase (MPO). As expected, at 6 hours post-infection with *K. pneumoniae*, lung MPO levels drastically increased (**Figure 3G**). The MPO activities in lung tissues of caspase-11 and NLRP3 deficient mice were significantly less compared to WT mice. Accordingly, the histology data indicated that at 6 hours post *K. pneumoniae* infection the lung tissue from WT mice demonstrated neutrophil infiltration and increased thickness of alveolar walls, which were milder in the lungs of caspase-11 and NLRP3 deficient mice (**Figure 4**).

### 3.7 Macrophages mediated bacterial clearance during *K. pneumoniae* infection

**Figure 5:**
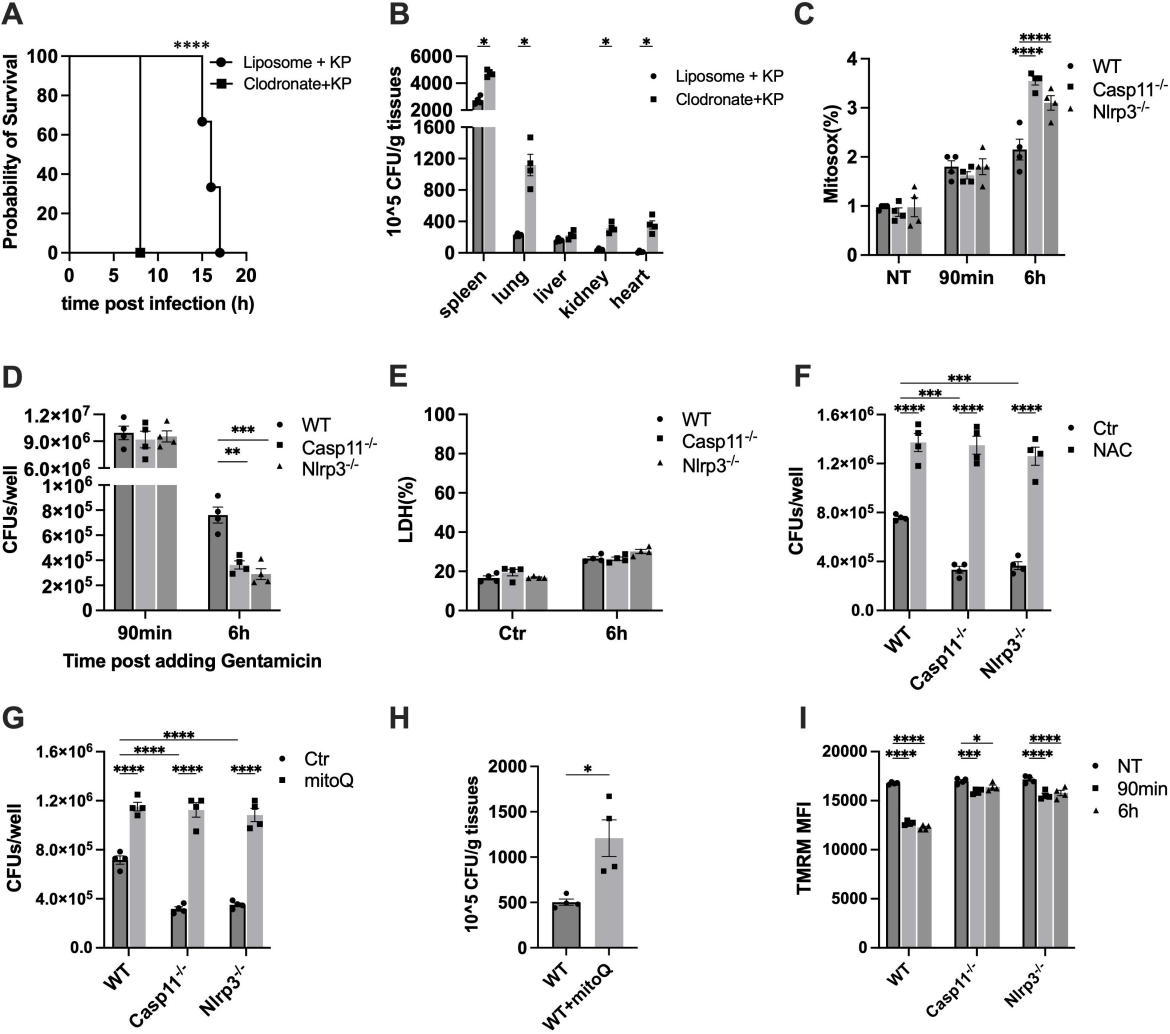
Macrophage mediated bacterial clearance during infection, in which mtROS played a role. **(A)** Depletion of macrophage significantly reduced mouse survival time. Mice were injected retro-orbitally with 40 mg/kg of liposomal clodronate at 24 h before being challenged with *K. pneumoniae* (1×10^6^ CFU per mouse) (Clodronate + KP). The same amount of plain liposomes was injected as control (Liposome + KP). Kaplan-Meier survival plots for mice are shown. n = 10-12 per group. ****p<0.0001 between Clodronate + KP group and Liposome + KP group by log rank test [Mantel-Cox]. **(B)** Depletion of macrophage increased bacterial burden in most key organs. Mice were injected (i.p.) with 5×10^6^ CFU of *K. pneumoniae* (KP). After 6 hours, mice were sacrificed and bacterial loads in the lung were determined. Error bars denote SEM; n = 4 for all experimental groups. *p<0.05 between Clodronate + KP samples and Liposome + KP samples by two-way ANOVA analysis. **(C-E)** Caspase-11 or NLRP3 deficiency led to increased mtROS production **(C)** and bacterial clearance **(D)**, but did not affect cell death **(E)** BMDMs from the indicated mice were treated with 50 MOI of *K. pneumoniae*. Mitosox were measured in non-treated (NT) and *K. pneumoniae* treated BMDMs at 90 minutes and 6 hours post addition via flow cytometer. Error bars denote SEM; n = 4 for all experimental groups. ***p<0.001, ****p<0.0001 between the WT and deficient strains by two-way ANOVA analysis. Intracellular *K. pneumoniae* were counted in BMDMs at 90 minutes and 6 hours post addition with gentamicin. Cell death was measured using the LDH assay at 6 hours post addition with gentamicin. Error bars denote SEM; n = 4 for all experimental groups. The statistical significance and comparisons among groups were analyzed by nonpaired t-test. **p<0.01, ***p<0.001. **(F, G)** Incubation with ROS scavenger compromised bacterial clearance in macrophages. BMDMs were infected with *K. pneumoniae* in the presence of NAC **(F)** or MitoQ **(G)** Intracellular *K. pneumoniae* were counted at 6 hours post addition with gentamicin. Error bars denote SEM; n = 4 for all experimental groups. The statistical significance and comparisons among groups were analyzed by nonpaired t-test. ***p<0.001, ****p<0.0001. **(H)** Pretreatment with MitoQ increased the bacterial load in the lung tissue. Mice were injected (i.p.) with MitoQ (0.1 mg per mouse) 48 hours before infection with *K. pneumoniae* as described. After 6 hours, mice were sacrificed and bacterial loads in the lung were determined. Error bars denote SEM; n = 4 for all experimental groups. *p<0.05 between macrophage depleted and control samples by two-way ANOVA analysis. **(I)** *K. pneumoniae*-infection led to mitochondrial membrane depolarization in BMDMs, which was alleviated by deficiencies in caspase-11 or NLRP3. BMDMs were incubated with 50 MOI of *K. pneumoniae*. MFI of TMRM was measured in non-treated (NT) and *K. pneumoniae* treated BMDMs at 90 minutes and 6 hours post infection via flow cytometer. Error bars denote SEM; n = 4 for all experimental groups. *p<0.05, ***p<0.001, ****p<0.0001 between WT and deficient strains by two-way ANOVA analysis.

To evaluate the role of macrophages in eliminating *K. pneumoniae* under our experimental condition, we depleted macrophages through retro-orbital injection of liposomal clodronate prior to inoculation with *K. pneumoniae* (32). Consistent with our previous reports, clodronate administration reduced blood monocytes by 90% within 24 h (data not shown) (18, 30). The survival of both control and macrophage-depleted mice following i.p. injection of *K. pneumoniae* was monitored. Macrophage depletion significantly shortened the survival time (**Figure 5A**). Subsequently, we measured the bacterial load in key organs at 6 hours post-injection (**Figure 5B**). As expected, the depletion of macrophages resulted in a significant increase in the bacterial burden in most key organs, indicating a critical role of macrophages in *K. pneumoniae* clearance.

### 3.8 Deficiency of caspase-11 or NLRP3 enhanced ROS production and bacterial killing

As ROS play a crucial role in bacterial killing, and macrophages are essential for bacterial clearance, we examined the impact of caspase-11 or NLRP3 on *K. pneumoniae*-induced mtROS production. BMDMs from WT mice or mice deficient in caspase-11 or NLRP3 were incubated with *K. pneumoniae*, and mtROS production was measured at 1.5 or 6 hours after incubation with bacteria as previously described (33). *K. pneumoniae* treatment promoted mtROS production, which was significantly enhanced in caspase-11^-/-^ and NLRP3^-/-^ BMDMs (**Figure 5C**), suggesting that caspase-11 and NLRP3 impaired ROS production upon *K. pneumoniae* infection in WT BMDMs.

To investigate whether caspase-11 and NLRP3 influence bacterial clearance in macrophages, we incubated BMDMs derived from WT, caspase-11^-/-^, or NLRP3^-/-^ mice with 50 MOI *K. Pneumoniae* for 90 min, allowing for bacterial internalization. Gentamicin was then added to kill extracellular bacteria. Cells were incubated for an additional 90 min or 6 hours. BMDMs were then harvested, and intracellular bacteria were counted (**Figure 5D**). The number of endocytosed bacteria at 90 min was similar in all three strains, suggesting that caspase-11 and NLRP3 deficiency did not affect the internalization of *K. pneumoniae*. At 6 hours, all macrophages contained significantly fewer bacteria, indicating effective clearance of intracellular *K. pneumoniae*. Caspase-11 or NLRP3-deficient macrophages were more effective in eliminating intracellular *K. pneumoniae*, as fewer bacteria were detected in these cells compared to WT cells. Under this experimental condition, the cell death at 6 h was similar among BMDMs from the WT and gene deficient strains (**Figure 5E**).

To further elucidate the role of ROS in mediating *K. pneumoniae* clearance, BMDMs from WT, caspase-11^-/-^, or NLRP3^-/-^ mice were treated with *K. pneumoniae* in the presence of a ROS inhibitor NAC, and intracellular bacterial load was measured as described 6 hours after the addition of gentamicin (**Figure 5F**). For all BMDMs, significantly higher numbers of bacteria were counted in NAC-treated group compared to the control group, strongly supporting a role of ROS in mediating the clearance of *K. pneumoniae* in macrophages. In addition, the bacterial loads in BMDMs from caspase-11^-/-^ or NLRP3^-/-^ mice were similar as in BMDMs from WT mice in the presence of NAC, indicating that the more efficient bacterial clearance observed in these knockout strains was abolished in the presence of a ROS scavenger.

To further elucidate the contribution of mitochondria ROS, we repeated the experiment using a more specific inhibitor for mtROS, MitoQ (**Figure 5G**). The result was very similar as in the case of NAC. The presence of MitoQ impaired bacteria clearance in all three BMDM samples, suggesting a vital role of mtROS in *K. pneumoniae* clearance. The differences between bacterial loads in the WT and caspase-11^-/-^ or NLRP3^-/-^ BMDMs were abolished after MitoQ treatment, indicating that the more efficient bacterial clearance in NLRP3^-/-^ or caspase-11^-/-^ BMDMs was mediated by mtROS.

Next, to evaluate the role of mtROS production during infection in *K. pneumoniae* clearance *in vivo*, we delivered MitoQ via i.p. injection at a dose of 0.1 mg per mouse 48 hours prior to bacterial inoculation. Bacterial load in the lung tissues was analyzed as described above after 6 hours (**Figure 5H**). Pre-treatment with MitoQ led to a significant increase of bacterial load in the lung tissues.

To investigate the possible mechanism of reduced mtROS production in caspase-11 and NLRP3 deficient BMDMs, mitochondrial accumulation of TMRM in WT, caspase-11^-/-^, and NLRP3^-/-^ was determined using flow cytometry, and the mean fluorescence intensity was measured as described (**Figure 5I**) (34–37). TMRM accumulation in mitochondria is sensitive to membrane potential, thus a reduction of the TMRM fluorescence intensity indicates mitochondrial membrane-depolarization. Incubation with *K. pneumoniae* led to a significant reduction of TMRM fluorescence in WT BMDM within 90 min, which persisted at 6 hours. Caspase-11 and NLRP3 deficiency diminished the reduction of TMRM fluorescence upon infection.

**Figure 6:**
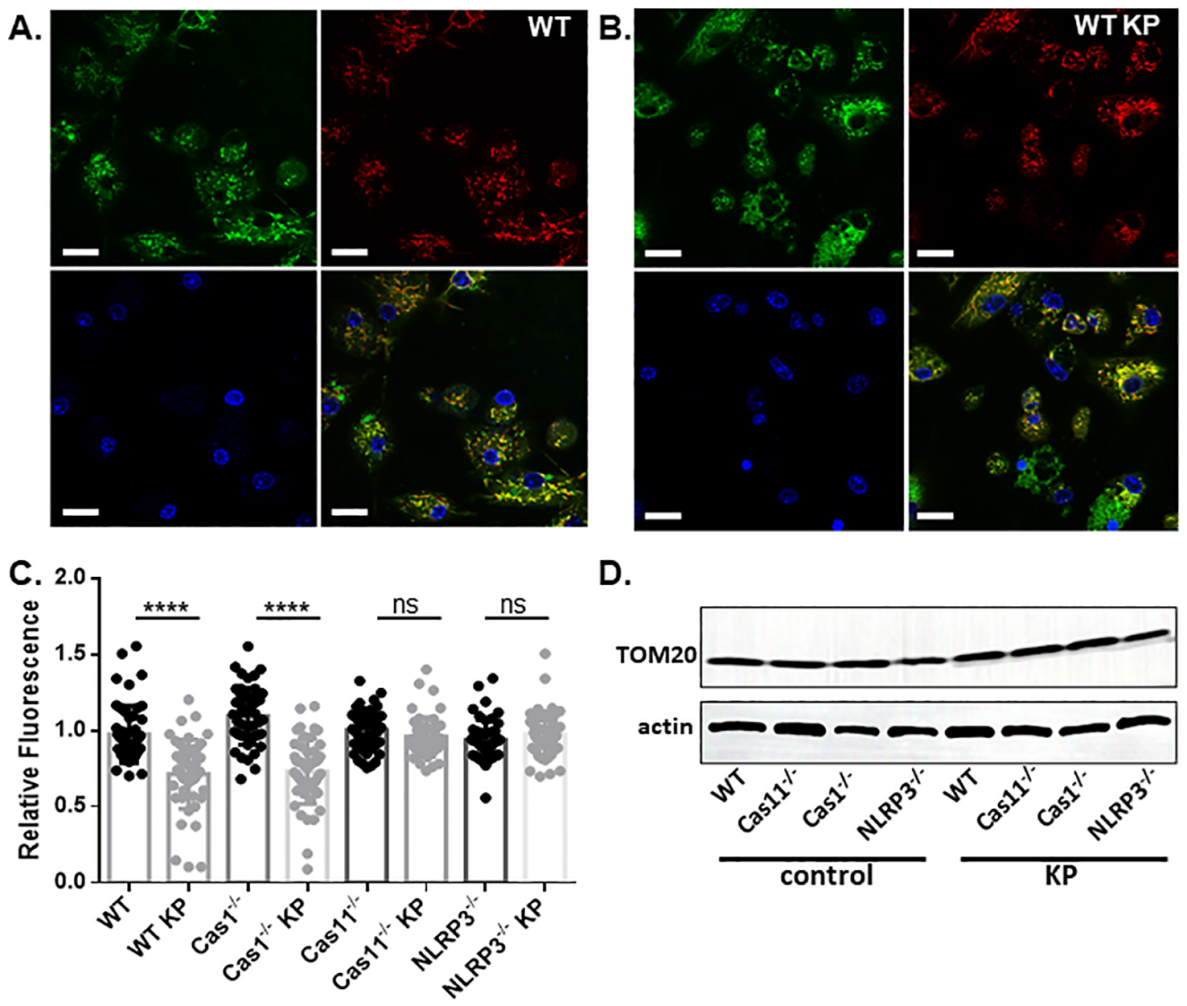
*K. pneumoniae* infection led to mitochondrial damage. A&B. Live cell imaging of BMDMs derived from WT mouse without **(A)** or with **(B)** treatment with *K. pneumoniae*. Cells were treated similar as described and stained with MTDR (red), MTG (green), and Hochest (blue). The last panel shows merged channels. The scale bar is 20 µm. **(C)** The ratio of the MTDR MFI over MTG MFI. ns no significance, ****p<0.0001 by unpaired t test. **(D)** Representative images of anti-actin and anti-TOM20 immunoblotting of BMDMs with or without *K. pneumoniae* treatment.

**Figure 7:**
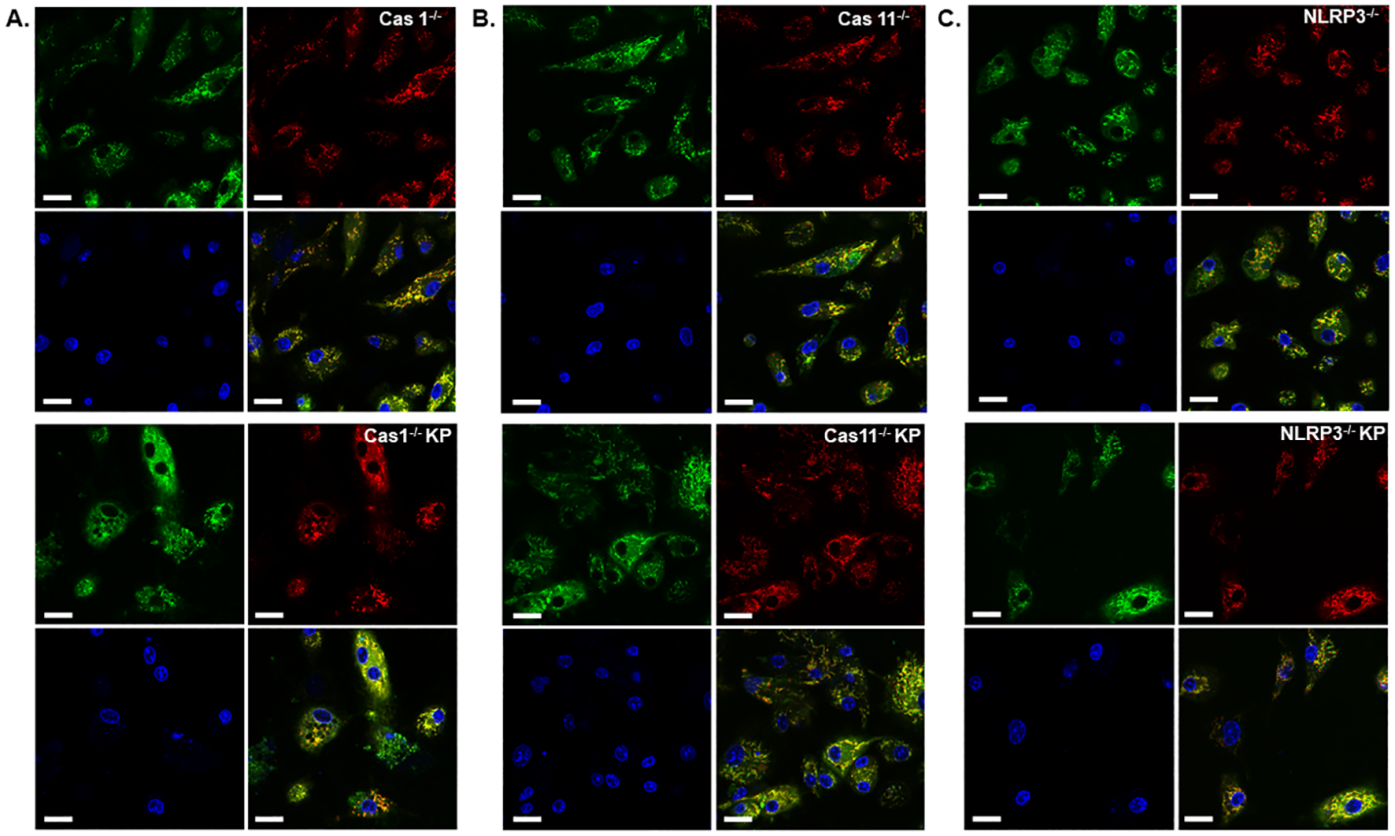
Deficiency of caspase-11 or NLRP3, but not caspase-1, diminished mitochondrial damage caused by *K. pneumoniae*. Live cell imaging of BMDMs derived from mice deficient in caspase-1 **(A)**, caspase-11 **(B)** or NLRP3 **(C)** without (upper panels) or with (lower panels) *K. pneumoniae* treatment. Cells were stained with MTDR (red), MTG (green), and Hochest (blue). The last panel shows merged channels. The scale bar is 20 µm.

Next, we conducted live cell imaging of mouse BMDMs incubated with *K. pneumoniae* to further examine mitochondrial damage. BMDMs were isolated from WT, caspase-1, caspase-11, or NLRP3 deficient mouse, and incubated with *K. pneumoniae* at 50 MOI. Gentamicin was added at 90 min after inoculation, and the cells were incubated for an additional 6 h. Mitochondria was then stained using MTG and MTDR. While accumulation of MTG in mitochondrial is not sensitive to the membrane potential, MTDR is a membrane potential-dependent fluorescence probe. The ratio of the mean fluorescence intensity MTDR/MTG was used to detect mitochondrial damage, as reduced accumulation of MTDR in mitochondria indicates malfunction. Nuclear was stained using Hoechst. As revealed in **Figures 6A, B**, incubation with *K. pneumoniae* led to a reduction of the MTDR/MTG ratio in WT BMDM (**Figure 6C**). BMDMs derived from mouse deficient in caspase-11 or NLRP3, but not caspase-1, were more resistant to *K. pneumoniae* treatment and significantly reduced mitochondrial damage were observed (**Figures 6C**, **7**). We also compared the abundance of mitochondria in each sample using immunoblotting to detect a mitochondria-specific protein marker, TOM20 (**Figure 6D**). No difference in the abundance of mitochondria were detected.

### 3.9 NLPR3 was critical for caspase-1 and caspase-11 activation in BMDMs upon infection with *K. pneumoniae*

**Figure 8:**
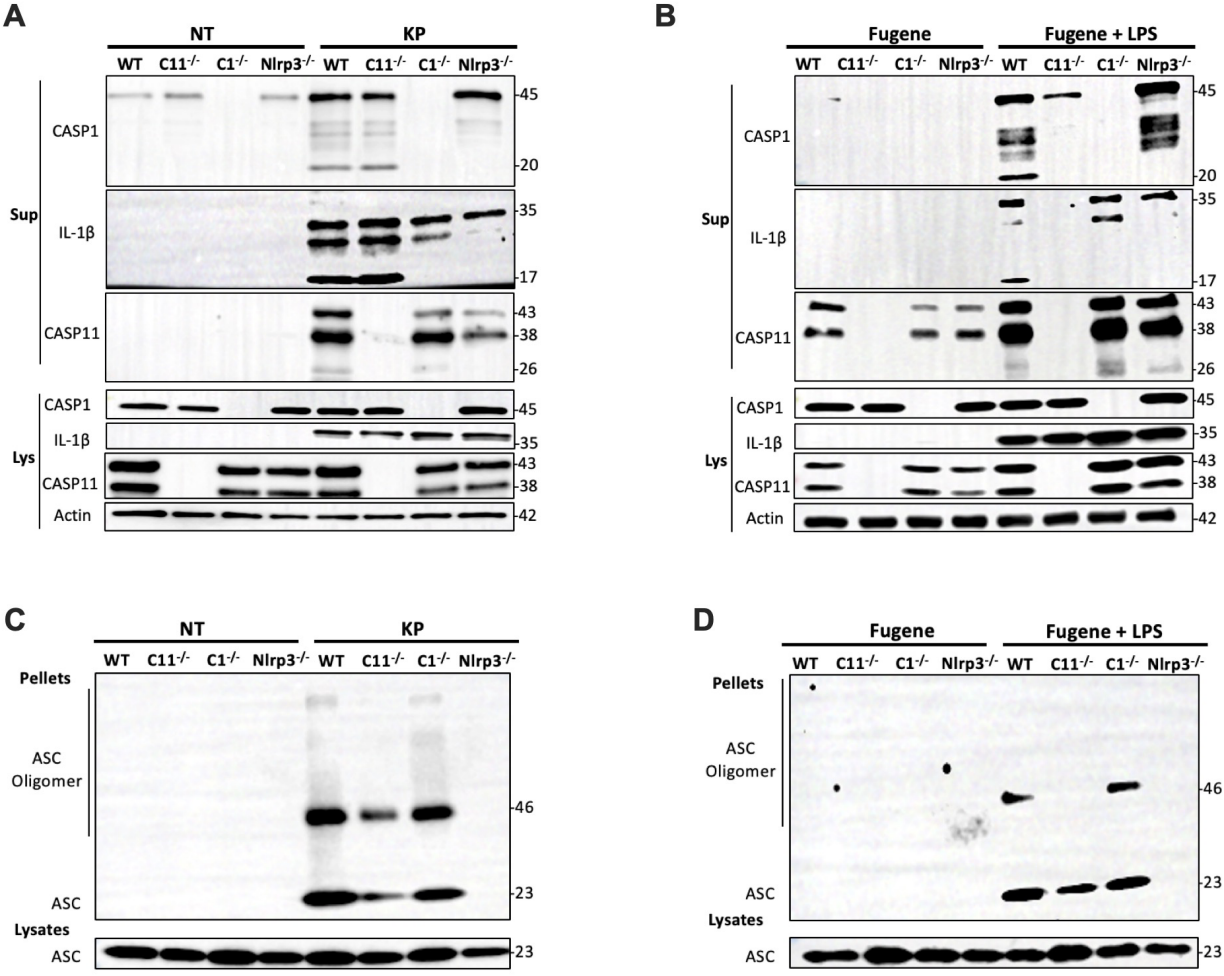
NLRP3 was critical for both caspase-1 and caspase-11 activation in BMDMs infected with *K. pneumoniae*. BMDMs were primed with 1 μg/mL Pam3CSK4 for 5 hours prior to the inoculation with 50 MOI of *K. pneumoniae* or the transfection with 2 μg/mL LPS via Fugene. **(A)** Representative immunoblot of caspase-1, IL-1β, caspase-11 and actin in the supernatant (Sup) and the lysate (Lys) from BMDM isolated from WT and caspase-11, caspase-1 or NLRP3 deficient mice 6 hours after the addition of *K. pneumoniae* (KP) or PBS (NT). **(B)** Representative immunoblot of caspase-1, IL-1β, caspase-11 and actin in the supernatant (Sup) and the lysate (Lys) from BMDM isolated from WT and caspase-11, caspase-1 or NLRP3 deficient mice 6 hours after the transfection of LPS via Fugene (Fugene + LPS) or Fugene alone (Fugene). **(C)** Representative immunoblot of ASC monomers and oligomers in the cross-linked pellets and soluble lysates from BMDM isolated from WT and caspase-11, caspase-1 or NLRP3 deficient mice 6 hours after inoculation with *K. pneumoniae* (KP) or PBS (NT). **(D)** Representative immunoblot of ASC monomers and oligomers in the cross-linked pellets and soluble lysates from BMDM isolated from WT and caspase-11, caspase-1 or NLRP3 deficient mice 6 hours after the transfection of LPS via Fugene (Fugene + LPS) or Fugene alone (Fugene). Chemical crosslinking was conducted by incubating the cell pellet with 2 mM disuccinimidyl suberate (DSS) at room temperature for 30 minutes.

To investigate the mechanism of NLRP3 inflammasome activation during *K. pneumoniae* infection, we conducted immunoblotting to monitor caspase-1 and caspase-11 cleavage in BMDMs (**Figure 8A**). BMDMs isolated from WT mice or mice deficient in caspase-1, caspase-11 or NLRP3 were incubated with *K. pneumoniae* for 6 hours. Supernatant from cell culture and the total cell lysate were analyzed for caspase-1 and caspase-11 activation and IL-1β cleavage. Caspase-1 cleavage was observed in the supernatant from WT and caspase-11 deficient BMDMs, but not NLRP3 deficient BMDMs, treated with *K. pneumoniae*, suggesting that NLRP3 but not caspase-11 played a role in caspase-1 activation. Accordingly, cleavage of IL-1β was observed in the supernatant from WT and caspase-11 deficient BMDMs, but not in caspase-1 deficient BMDMs. Caspase-11 cleavage was observed in the supernatant from WT and caspase-1 deficient BMDMs, but not in NLRP3 deficient BMDMs. These data suggest that activation of caspase-11 required NLRP3 and was partially dependent on caspase-1. Thus, NLRP3 seemed to be upstream of both caspase-11 and caspase-1 during *K. pneumoniae* infection. LPS is a virulence factor in *K. pneumoniae* and is known to induce the cleavage of caspase-1, caspase-11 and IL-1β through a noncanonical pathway (38–40). Therefore, we transfected LPS using Fugene for 6 hours and performed similar immunoblotting (**Figure 8B**). As expected, cleaved caspase-1 and IL-1β were found in the supernatant from WT BMDMs treated with LPS/Fugene, but not in caspase-11 and NLRP3 deficient BMDMs, indicating that both caspase-11 and NLRP3 were necessary for caspase-1 activation and IL-1β cleavage upon LPS treatment. The activation of caspase-11 was detected in the supernatants from WT, caspase-1 and NLRP3 deficient BMDMs, indicating that caspase-11 activation was independent of both caspase-1 and NLRP3.

We further examined ASC oligomerization, an indicator of inflammasome activation, in BMDMs deficient in caspase-1, caspase-11, or NLRP3 upon infection with *K. pneumoniae*. ASC oligomerization in the cell pellets were analyzed after crosslinking using disuccinimidyl suberate (DSS) (**Figure 8C**). ASC oligomers were observed in caspase-1 and caspase-11 deficient BMDMs, indicating that deficiency in neither caspase-1 nor caspase-11 abolished the activation of NLRP3. In contrast, upon LPS transfection, ASC oligomers were detected in WT and caspase-1 deficient BMDMs, but not in caspase-11 deficient BMDMs, indicating that caspase-11 but not caspase-1 played a role in LPS induced NLRP3 activation (**Figure 8D**). These data further demonstrated that NLRP3 acts upstream of both caspase-11 and caspase-1 during *K. pneumoniae* infection, which differs from the mechanism of intracellular LPS induced inflammasome activation.

## 4 Discussion

*K. pneumoniae* infection has been the focus for many studies, but most of them used the lung infection model through intranasal or intratracheal delivery (41–47). Among the few studies using the i.p. injection mouse model, the lethal dosages varied drastically, likely due to differences in the mouse and/or bacteria strains used. Franklin et al. reported that *K. pneumoniae* (strain 43816 serotype 2) at a low dose of 100 CFU induced the death of Swiss Webster mice in 5 days, and 10^4^ CFU kill mice within 24 hours (48). Similar as reported here, *K. pneumoniae* was found accumulated at high levels in the lung. In a study using C57BL/6, *K. pneumoniae* (strain 467) at the dosage of ~10^4^ led to death in 5.5~6 days (49). In another study using C57BL/6, a hypervirulent *K. pneumoniae* strain (SGH10) at the dosage of 10^5^ CFU per mouse did not lead to death at 30 h when mice were euthanized (50). In this study, we used C57BL/6J mouse, and found that at dosage of 10^6^ CFU per mouse, all mice died within 20 hours. We examined the potential mechanisms that are known to lead to acute death, including coagulation, inflammation, and lung failure. We found that *K. pneumoniae* rapidly led to acute lung function failure. We also found *K. pneumoniae* delivered via i.p. injection accumulated quickly in the lung. The NLRP3/caspase-11 pathway serves as a crucial mechanism that limits bacterial clearance during *K. pneumoniae* infection by macrophages.

Caspase-11 mediates LPS-triggered inflammasome activation and pyroptosis through the non-canonical inflammasome activation pathway (38–40, 51). Since LPS is an important virulence factor of *K. pneumoniae*, the contribution of caspase-11 to *K. pneumoniae*-evoked pneumonia has been investigated (12, 13). Bacteria were inoculated through intranasal administration in these studies. Wang et al. found (12) that deficiency of caspase-11 led to significantly higher lung bacterial load, lower serum IL-1α secretion, more severe lung damage, and increased serum level of IL-6 and TNFα. These findings suggest that caspase-11 plays a role in the neutrophil recruitment and bacterial clearance in the lung infection model. Perlee et al. (13) also reported that *K. pneumoniae* load increased in the capase-11 deficient mice. However, they did not observe any effect of caspase-11 deficiency on neutrophil recruitment or bacterial dissemination to other organs. In addition, despite of the higher bacterial load, no impact on lung pathology was observed in the *K. pneumoniae* lung infection model.

We used a systemic infection model via intraperitoneal inoculation. We believe that this model of *K. pneumoniae* infection is (patho)physiologically relevant, as gastrointestinal tract serves as a major reservoir for *K. pneumoniae* (15). Thus, understanding the mechanism of pathogenesis by *K. pneumoniae* systemic infection is of significance. In contrast to the lung local infection model, we observed that caspase-11 deficiency protected against lung injury. In consistent with this finding, caspase-11 deficiency significantly enhanced bacterial clearance in lungs and prolonged mouse survival time. While intraperitoneal injection mouse model is a widely used experimental method to study infections, we acknowledge that there are several limitations. As we have discussed, the peritoneal might not be the most physiological route of infection for *K. pneumoniae*, which may result in immune responses that differ from those initiated by other routes (e.g., respiratory). Intraperitoneal inoculation may bypass mucosal barriers and epithelial barriers, which could exaggerate their virulence compared to natural infection routes. The peritoneal cavity is rich in macrophages and other immune cells, which may induce an inflammatory response that does not reflect systemic or localized immune responses observed in natural infections.

It is interesting that the pulmonary IL-6 level, but not the IL-1β or TNFα levels, significantly decreased in mice deficient in caspase-11 or NLRP3 upon infection (**Figure 3D-F**). The level of IL-6 is influenced by various factors, including IL-1β and TNFα (52). During infection, transcription can be upregulated by pathogen-associated molecular patterns (e.g., LPS) and pro-inflammatory cytokines. Additionally, numerous RNA-binding proteins and microRNAs have been identified as posttranscriptional regulators of IL-6 mRNA stability (53–55). Therefore, the observed discrepancy between IL-6 and IL-1β/TNFα levels in different strains likely resulted from the combined effects of these diverse regulatory factors.

Depletion of macrophages by pre-injection of clodronate significantly increased bacterial load (in lungs), as well as shortened survival times. It appears that in our i.p. injection model, macrophages play a crucial role in killing *K. pneumoniae* during its trafficking from the local infection (injection) site to blood and then various organs, and caspase-11 deficiency might enhance the function of macrophages in bacterial clearance. This may explain the discrepancy in the role of caspase-11 in *K. pneumoniae* infections between systemic and local lung infection models because bacterial trafficking is less relevant in the local infection model. A recent study reported the inactivation of neutrophils in mice upon injection of clodronate liposome (56). Using a mouse model of K/BxN serum transfer arthritis, the authors found that the “stunning” of neutrophil function, rather than depletion of macrophage/monocyte, was responsible for the anti-inflammatory effect of clodronate liposome in mouse. Here we can’t rule out the potential contribution of neutrophils in the increase of bacterial load in lung tissues and reduction of mouse survival after clodronate liposome treatment.

It is well known that mtROS is an important mechanism of eliminating intracellular bacteria (57). Incubation of mouse BMDMs with *K. pneumoniae* indeed significantly increased mtROS production, which was further enhanced in the caspase-11 or NLRP3 deficient BMDMs, correlating with enhanced clearance of endocytosed bacteria. mtROS production and bacterial killing were elevated in caspase-11 or NLRP3 deficient BMDM, suggesting a role of these proteins in disrupting mitochondria function upon *K. pneumoniae* invasion. This conclusion is supported by the data that incubation of mouse BMDMs with *K. pneumoniae* induced disruption of the mitochondrial membrane potential through caspase-11 and NLRP3, because their deficiency protected against *K. pneumoniae*-induced mitochondria damage. Interestingly, caspase‐11 has been reported to counteract mtROS‐mediated clearance of *Staphylococcus aureus*, a Gram-positive pathogen, in murine macrophages (58). *S. aureus* induces higher mtROS production in caspase-11 deficient than in WT macrophages, similar to our observation. In terms of mechanism, association of *S. aureus*-containing vacuoles with mitochondria was found increased in caspase-11^-/-^ macrophages. In another study, *Legionella pneumophila* survival *in vivo* and *in vitro* was found increased in the caspase-11 deficient mice and their derived macrophages (59). The underlying mechanism was proposed to be through a role of caspase-11 in regulating actin polymerization, which affected fusion of vacuoles with lysosome.

The role of NLRP3 in bacterial infection has been intensively investigated (60–62). The well-accepted concept is that intracellular LPS activates caspase-11 through direct binding to caspase-11 (40), and then activates NLRP3 through several different mechanisms (60). Activation of NLRP3 leads to caspase-1 cleavage and activation, which subsequently cleaves pro-IL-1β and pro-IL-18 as well as GSDMD, resulting in IL-1β and IL-18 secretion and cell pyroptosis. Thus, activation of caspase-11 is the initial step of the signaling cascade triggered by LPS stimulation, as LPS-induced caspase-1 cleavage, as well as ASC speck formation, were abolished in the caspase-11 deficient BMDMs (**Figure 8B, D**). Interestingly, *K. pneumoniae*-induced caspase-1 and -11 cleavage was abolished in the NLRP3 deficient BMDMs (**Figure 8A, C**). Therefore, instead of downstream from caspase-11, NPRP3 is upstream of caspase-11 activation during *K. pneumoniae* infection. Further studies are needed to decipher how *K. pneumoniae* activates NLRP3 and how NLRP3 inflammasome regulates caspase-11 activation in macrophages during *K. pneumoniae* infection.

## Data Availability

The raw data supporting the conclusions of this article will be made available by the authors, without undue reservation.
